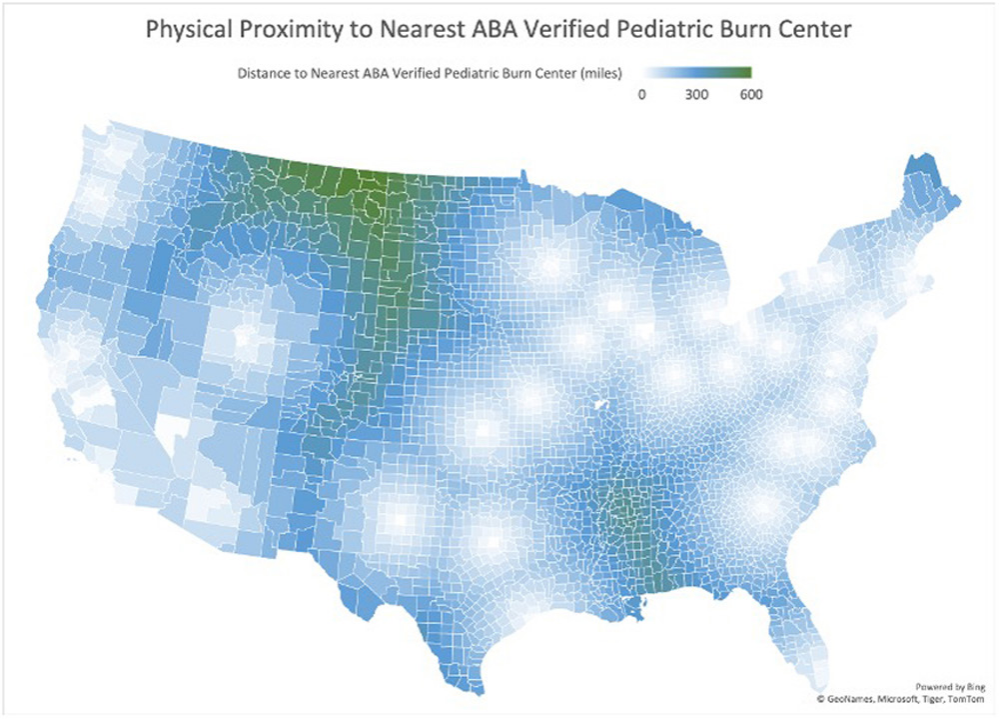# 39 Certified Burn Center Access in the United States: The Geospatial and Transport Cost of Transfer

**DOI:** 10.1093/jbcr/irad045.013

**Published:** 2023-05-15

**Authors:** Shelly Edwards, Madyson Brown, Laura Humphries, Ian Hoppe

**Affiliations:** University of Mississippi Medical Center, Jackson, Mississippi; University of Mississippi Medical Center, Jackson, Mississippi; University of Mississippi Medical Center, Division of Plastic and Reconstructive Surgery, Jackson, Mississippi; University of Mississippi Medical Center, Division of Plastic and Reconstructive Surgery, Jackson, Mississippi; University of Mississippi Medical Center, Jackson, Mississippi; University of Mississippi Medical Center, Jackson, Mississippi; University of Mississippi Medical Center, Division of Plastic and Reconstructive Surgery, Jackson, Mississippi; University of Mississippi Medical Center, Division of Plastic and Reconstructive Surgery, Jackson, Mississippi; University of Mississippi Medical Center, Jackson, Mississippi; University of Mississippi Medical Center, Jackson, Mississippi; University of Mississippi Medical Center, Division of Plastic and Reconstructive Surgery, Jackson, Mississippi; University of Mississippi Medical Center, Division of Plastic and Reconstructive Surgery, Jackson, Mississippi; University of Mississippi Medical Center, Jackson, Mississippi; University of Mississippi Medical Center, Jackson, Mississippi; University of Mississippi Medical Center, Division of Plastic and Reconstructive Surgery, Jackson, Mississippi; University of Mississippi Medical Center, Division of Plastic and Reconstructive Surgery, Jackson, Mississippi

## Abstract

**Introduction:**

Specialized burn centers are critical in minimizing burn-associated morbidity and mortality. However, American Burn Association (ABA)-verified burn centers are unequally distributed across the US, and fewer verified centers are available for pediatric patients relative to adults. The economic burden of transport to verified centers represents a significant proportion of the already high cost of burn-associated care. The present study aims to quantify inequitable burn care access in the contiguous US due to age group and locality as a function of physical proximity and transportation cost.

**Methods:**

County-level distances (n=3,106) to the nearest ABA-verified adult or pediatric burn center were determined and mapped. Distances were then analyzed separately for rural (n = 1440) and urban (n = 1666) counties for both adult and pediatric burn centers. Distance calculations for each population were combined with transport cost data (2022 CMS Ambulance fee Schedules) to determine the average cost of transport for each patient population (adult vs. pediatric, urban vs. rural).

**Results:**

59 Adult and 43 Pediatric ABA-verified centers were identified from the ABA burn center directory. Pediatric patients reside 30.57 miles (p < 0.001) further than adults from the nearest center, accounting for a 10.5% - 15.9% transport cost increase. Transport costs increased dramatically between urban and rural counties, with rural patients facing a cost increase of 32.7% and 80.88% for ground and air transportation, respectively.

**Conclusions:**

Physical proximity to burn care may appear to differ only modestly across age and region. However, the seemingly marginal increase in distance significantly impacts the cost of patient transport. The present study highlights both physical and economic barriers to burn care access faced by rural and pediatric patients. Increasing ABA burn center certification in targeted areas across the US may decrease the disparities in access to burn care faced by these groups.

**Applicability of Research to Practice:**

Identify areas that may benefit from expansion of certified burn-care access and additional provider education.